# From trial to practice: incidence and severity of COVID-19 vaccine side effects in a medically at-risk and vaccine-hesitant community

**DOI:** 10.1186/s12889-022-14824-z

**Published:** 2022-12-14

**Authors:** Melinda C. Joyce, Natalie J. Mountjoy, Julia A. Johnson, John T. Newman, David L. Bandy, Nasri A. Atalla, Aniruddha Singh, Doug McElroy

**Affiliations:** 1 Western Kentucky Heart and Lung/Med Center Health Research Foundation, 421 U.S. 31W Bypass, Bowling Green, KY 42101 USA; 2 Department of Biology, Western Kentucky University, 1906 College Heights Boulevard #11008, Bowling Green, KY 42101-1080 USA; 3 University of Kentucky College of Medicine – Bowling Green Campus, 399 U.S. 31W Bypass, Bowling Green, KY 42101 USA; 4 Centre College, 600 West Walnut Street, Danville, KY 40422 USA; 5 Cardiovascular Fellowship, Reading Hospital, 420 S. 5th Avenue, West Reading, PA 19611 USA

**Keywords:** COVID-19, Vaccine side effects, BNT162b2, mRNA-1273, Vaccine hesitancy

## Abstract

**Background:**

The rapid authorization and widespread rollout of COVID-19 vaccines in the United States demonstrated a need for additional data on vaccine side effects, both to provide insight into the range and severity of side effects that might be expected in medically-diverse populations as well as to inform decision-making and combat vaccine hesitancy going forward. Here we report the results of a survey of 4825 individuals from southcentral Kentucky who received two doses of either the Pfizer-BioNTech (BNT162b2) or Moderna (mRNA-1273) vaccine between December 14, 2020 and May 1, 2021. As new versions of the vaccine are rolled-out, local initiatives such as this may offer a means to combat vaccine hesitancy in reference to COVID-19, but are also important as we face new viral threats that will necessitate a rapid vaccine rollout, and to combat a growing public distrust of vaccines in general.

**Methods:**

Individuals that received two doses of either BNT162b2 or mRNA-1273 between December 14, 2020 and May 1, 2021 were sent a survey, created by the research team. Respondents were asked to rate the incidence and severity of 15 potential side effects and two related outcomes following each of their two doses of the vaccine. All statistical analyses were carried out using SYSTAT, version 13. The data were analyzed utilizing a range of statistical tests, including chi-square tests of association, Cohen’s h, Kruskal-Wallis test one-way nonparametric ANOVA, least-squares regression, and Wilcoxon signed-ranks test. Significance was assessed using Bonferroni-adjusted criteria within families of tests.

**Results:**

In general, the pattern and severity in side effects was similar to both clinical trial data as well as other published studies. Responses to the mRNA-1273 vaccine were more severe than to BNT162b2, though all were generally in the mild to moderate category. Individuals who reported having previously tested positive for COVID-19 reported stronger responses following the first dose of either vaccine relative to COVID-naïve individuals. The reported severity to the COVID-19 vaccine was positively correlated with self-reported responses to other vaccines.

**Conclusions:**

Our findings allow broad-scale estimates of the nature and severity of reactions one might expect following vaccination within a clinically-diverse community, and provide a context for addressing vaccine hesitancy in communities such as ours, where locally-generated data and communication may be more influential than national trends and statistics in convincing individuals to become vaccinated. Further, we argue this community-based approach could be important in the future in three key ways: 1) as new boosters and modified vaccines re-volatilize vaccine hesitancy, 2) as new vaccines receive similar testing and rapid authorization, and 3) to combat vaccine hesitancy in other arenas (e.g., annual vaccines, childhood vaccines).

**Supplementary Information:**

The online version contains supplementary material available at 10.1186/s12889-022-14824-z.

## Background

On December 11, 2020, the U.S. Food and Drug Administration (FDA) approved Emergency Use Authorization (EUA) for the Pfizer-BioNTech mRNA-based COVID-19 vaccine (BNT162b2). A second EUA was issued for Moderna’s version of the vaccine (mRNA-1273) one week later, on December 18, 2020. Following subsequent review and approval of each vaccine by the Centers for Disease Control (CDC), the first vaccinations outside of clinical trials were given to U.S. citizens on December 14 and 21, respectively.

Emergency Use Authorization was based on data from Phase III clinical trials, including reactogenicity profiles generated from subsets of the clinical data. Profiles of vaccine reactogenicity [1] to BNT162b2 vaccine were based on an initial sample of 4093 healthy individuals from one of two age groups – 18 to 55 years, and 55+ years of age [2]. The mRNA-1273 trial utilized a similar design, encompassing 15,163 healthy individuals falling into either the 18 to 64 or 65+ years of age group [3]. Trials of both vaccines generated data suggesting high safety and efficacy, with typical and mild local and/or systemic reactions. The most common reactions reported in both trials were pain, redness, and swelling at the injection site, fever, headache, fatigue, chills, myalgia, and nausea/vomiting. In the mRNA-1273 trial, axillary swelling/tenderness was reported by 10–14% of individuals [3]. Both vaccines elicited more frequent and stronger reactions following the second dose of the two-dose series [2, 3]. Both showed lower incidence of systemic reactions than non-mRNA counterparts [4].

Typically, vaccine development takes years [5], encompassing multiple clinical testing phases; however, the COVID-19 vaccine review process was expedited given the severity of the pandemic [6]. EUAs of both vaccines were issued within approximately 9 months of initiation of Phase 1 trials in March, 2020 [7]. While subsequent trials and analyses of data from mass rollouts of the vaccine have confirmed their safety and efficacy, the speed of the approval process – while justified by the urgent need to combat the pandemic – generated skepticism and/or fear among some segments of the public that has led to vaccine hesitancy. Intentional or unintentional dissemination of misinformation has exacerbated this concern [8].

A survey of more than 55,000 adults in April/May 2021 [9, 10] found that the most cited reasons for COVID-19 vaccine hesitancy were: concern about possible side effects (52%); mistrust of government (45%); desire to wait and see if the vaccine is safe (35%); and concern about the vaccine’s effectiveness (24%). These studies partitioned hesitancy by education level, employment, age, ethnicity, and political affiliation. In general, hesitancy was higher among older and less educated individuals, and those living in a rural counties or ones with a higher Republican vote in the 2020 presidential election [9, 10]. A scoping review of the literature identified similar trends in multiple countries [11].

Unfortunately, these initial concerns have not attenuated over time. If anything, hesitancy and/or complacency has only grown as the world has experienced recurrent variant-driven surges in COVID infection, and benefitted from development of multiple vaccine boosters. Although the wide availability of vaccines in the developed world may appear to have attenuated COVID risk, the vaccine rate has slowed in the U.S. [12], and a recent meta-analysis showed vaccine hesitancy still exists in the U.S. at a rate of 43.5% [13]. Meanwhile, the pandemic has begun to enter an endemic state [14].

Vaccine hesitancy is also not unique to COVID-19 [15, 16]. We are currently experiencing a “Vaccine-Hesitant Moment” [17], partially exposed by the public response to the COVID-19 vaccine, but also problematic across vaccination types (e.g., measles–mumps–rubella (MMR), human papillomavirus (HPV), seasonal flu (H1N1, etc.). A recent review [18] cites the increasing power of digital platforms and extremism, declining public trust in expertise, shifting preferences for alternative health, and deepening political polarization as drivers of this trend.

The COVID-19 pandemic elucidated both the magnitude and potential hazard of this vaccine-hesitant moment. As of June 2022, there have been more than 500 million confirmed cases of COVID-19 and 6.3 million deaths globally [19]. Although vaccines have been available in some countries for more than a year, global vaccination rates – with less than 50% fully vaccinated and boosted – show a significant portion remain unvaccinated [12]. A recent model showed the potential for a seven-times higher mortality rate in countries where vaccine hesitancy remains problematic [20].

Evidence of this continued hesitancy points to the need for additional and accumulative data that address public concerns regarding COVID-19 vaccines. In addition to generating data on a wider range of patients – including those with comorbidities that impact their susceptibility to COVID-19 or response to vaccination – such data allow individuals to use more localized information in their decision-making regarding vaccination and its likely side effects [21].

In the context of COVID-19, Kentucky is an ideal source population for study, as it sits squarely at the intersection between clinical and social risk [22]. Kentucky ranks 36th for adult preventive services; 37th for annual immunizations; 49th for adult smoking; 45th for diabetes and obesity; 48th for cancer and cardiovascular disease; and 49th for chronic obstructive pulmonary disease [23]. These comorbidities increase susceptibility to severe COVID-19 disease [24–26]. Vaccine hesitancy is also present in the Commonwealth. Despite strong advocacy from the Governor, state officials, and the healthcare community – Kentucky, a rural, undereducated, Republican-leaning state, lags behind the national average in percentage of its eligible population who are vaccinated [27, 28]. As of Fall 2022 – nearly 2 years after public vaccination programs began – only 58.2% of the population have completed the initial vaccination series, and only 42.0% have received one booster dose [29], far from the 75–85% often cited for herd immunity [14], and 10% less than the national average. These factors suggest Kentuckians remain both more at-risk from COVID-19 [24] and more likely to underestimate or minimize that risk [9, 10].

Here we report the results of a survey of nearly 5000 recipients of either the BNT162b2 or mRNA-1273 vaccine series. Respondents were asked to identify and rate the severity of side effects experienced following their first and second doses. Using these data, we ask three specific questions: (1) How do our findings on side effects following vaccination compare to those seen in clinical trials and other published studies; (2) What is the severity and pattern of side effects seen in respondents who reported a previous diagnosis of COVID-19 relative to COVID-19 naïve respondents; and (3) What is the relationship between severity of side effects from the COVID-19 vaccine and those resulting from other vaccines?

We discuss our results in the context of addressing ongoing COVID-19 vaccine hesitancy in local, high-risk populations and how our community-based model, illustrating vaccine safety, may be transferred to a wider context in three key ways: 1) as new boosters and modified vaccines re-volatilize vaccine hesitancy, 2) as new vaccines receive similar testing and rapid authorization, like in the case of Monkey Pox, 3) and, to combat vaccine hesitancy in other arenas (i.e., annual vaccines, childhood vaccines, etc.).This holds true as various boosters become available and the vaccine is adapted for newly evolved strains of COVID-19, and is also relevant outside the current context of COVID-19 as we look ahead at the next viral outbreak. In a wider context, hesitancy regarding other vaccines (e.g., seasonal flu and childhood vaccines) is becoming an increasing issue across the U.S. [30, 31].

## Methods

### Study location and population

Med Center Health is a six-hospital system serving over 280,000 individuals across ten counties in southcentral Kentucky. Within three days of COVID-19 vaccine authorization, Med Center Health established a vaccine clinic at its main campus in Bowling Green, Kentucky. As vaccine became more plentiful, clinics were established at four other hospitals within the system. At the peak of vaccine rollout, these facilities collectively administered approximately 900 doses per day (M. Joyce, pers. comm.). Over the study period, more than 41,380 first doses of COVID-19 vaccine were administered.

Med Center Health followed the four-phase vaccine rollout plan developed and stipulated by Kentucky Governor Andy Beshear, in accordance with national and state public health officials [32]. Because of patterns of vaccine availability and distribution, most individuals received the BNT162b2 vaccine, while somewhat fewer received mRNA-1273; a small number received the Janssen (JNJ-78436735) vaccine.

### Sample

We deployed an online survey to individuals who received at least one vaccination through Med Center Health between December 14, 2020 and May 1, 2021. Med Center Health staff compiled a cell phone contact list of 18,711 such individuals, who were each sent a survey invitation via text message on May 5, 2021. The invitation, which included a link to the online survey, was sent successfully to 17,760 devices. Text reminders were sent on May 12 and June 12. On June 12, we texted the survey invitation to an additional 1830 individuals vaccinated after May 5, bringing the total pool to 19,590 potential respondents. One final reminder was sent to the entire list on June 18 and the survey closed on June 21, 2021.

### Instrument

Data were collected anonymously following Dillman’s Tailored Design Method [33] for internet surveys. The questionnaire was optimized for mobile viewing [34] and consisted of several pathways. The various pathways were pilot tested among 24 college students, office staff, pharmacists, medical interns and physicians for validity, clarity, timing and mobile layout.

The survey questionnaire was developed by the research team in Qualtrics and approved by the Medical Center IRB #1. All respondents provided informed consent in order to enter the questionnaire and all collected response data were kept on a secure device.

Respondents were first asked a series of demographic questions, followed by general information on their vaccine status and which vaccine type they had received. The majority of remaining question items were identical to those used in clinical vaccine trials, for the BNT162b2 [2] and mRNA-1273 [3] vaccines. These large question blocks asked respondents to rate the severity of 15 potential side effects. Severity rating categories were Likert-based and included: none (1; no symptoms), mild (2; did not interfere with activities), moderate (3; interfered with some activities), severe (4; prevented regular daily activity), and medical care required (5; ER, doctor, or hospital).

Additionally, respondents were asked to rate the severity of any unsolicited adverse events they experienced, if they required medications to treat their side effects, and the number of days missed from work or other activities as a result of side effects. They were also asked to rate their overall reactivity to other common vaccines (e.g., flu, pneumonia, tetanus and shingles), if they had been diagnosed with COVID-19 prior to vaccination and if so, how long ago in reference to their vaccine date.

### Statistical analyses

All statistical analyses were carried out using SYSTAT, version 13. Because of sample size limitations, statistical comparisons were restricted to respondents who received two doses of the BNT162b2 or mRNA-1273 vaccine.

Individual items were tested for significant differences in level of side effects reported between vaccine brands using chi-square tests of association, separately for first and second doses. Overall side effect severity scores were calculated for each respondent as the mean response level across all listed side effects; separate severity scores were computed for first-dose and second-dose reactions.

Side effects incidence rates were compared to those reported from clinical trials data via calculation of incidence rate ratios (IRR). Statistical significance of IRRs was determined based on 95% confidence intervals, and clinical significance was qualitatively assessed using Cohen’s h [35].

Differences in severity scores by age and gender were tested for significance using a Kruskal-Wallis test, and relationships between age and severity scores were estimated using least-squares regression. Differences between BNT162b2 and mRNA-1273 vaccines were tested for significance using Kruskal-Wallis one-way nonparametric ANOVA. Differences between first- and second-dose severity scores were examined using a Wilcoxon signed-ranks test applied separately between vaccine types. Differences between respondents who reported previously tested positive for COVID-19 vs. being COVID-19 naïve were tested using a Kruskal-Wallis test. Correlations between overall severity scores for both first and second doses of each COVID-19 vaccine type were compared to respondents’ self-reported severity of reactions to vaccines for influenza, pneumonia, tetanus, and shingles using Pearson correlation coefficients. Significance of all comparisons was based on Bonferroni-adjusted criteria applied within families of tests.

## Results

The initial response rate for our survey was 29% (*n* = 5738), with a 96% consent rate (*n* = 5525). A small percentage of respondents indicated they had not received the vaccine (1.0%), could not remember which vaccine they received (1.1%), received the JNJ-78436735 vaccine (0.5%), or had not completed their vaccine course (2.5%). Of the latter, the majority (84.9%) identified scheduling issues as the reason, while smaller percentages indicated it was not yet their turn (6.7%) or that they had concerns about vaccine safety (6.7%). Deletion of these and other incomplete responses resulted in a pool of 4825 respondents who had received both doses of either the BNT162b2 or mRNA-1273 vaccine.

### Respondent characteristics

Respondents were predominantly female (73.1%) and identified as Caucasian (91.8%). The median age group was 56–60 years. The largest number of respondents were 61–65 years of age (13.7%), and greater than 50% of respondents were aged 51–75 (55.2%). Healthcare workers made up the largest single group of respondents (28.9%), followed by individuals aged 70 and older (18.5%), 60–69 years of age (15.2%), K-12 school personnel (10.4%), adults aged 15–59 (7.8%), and essential workers (7.0%). These demographic characteristics mirror the ethnic makeup of the community from which the sample was drawn [36], combined with Kentucky’s phased rollout of the vaccine during the timeframe of the study [32]. Complete respondent characteristics are given in Table 1.

**Table 1:** Respondent Characteristics. Shown are data for the 4825 respondents who received two doses of either the BNT162b2 (Brand = P) or mRNA-1273 (Brand = M) vaccine. Acronyms for Phase categories are as follows: HC = healthcare workers; 1st R = first responders; 65+ = adults age 70 or older, K12 = school personnel; CC = child care workers; EW = essential workers; 16 + H = individuals age 16 or older with underlying health conditions; 60–69 = adults age 60–69; and 15–59 = individuals age 50–59. Categories of the most important factor behind respondents’ decision to get the vaccine are as follows: Self = to protect oneself; 2 = Others = to protect others; Activities = to resume activities; Masks = to reduce the need for masks. The sample size (N) of respondents indicating whether they had ever received other vaccines was 4786. Of respondents who indicated they had been previously diagnosed with COVID-19, the response categories represent the number of days prior to receiving their first dose of either the BNT162b2 or mRNA-1273 vaccine

Gender – no. (%)	Male	Female	Non Binary	Other			
	1283 (26.6)	3525 (73.1)	11 (0.2)	6 (0.1)			
Ethnicity – no. (%)	Asian/PI	Afr. Amer.	Hispanic	Nat. Amer.	Caucasian	2 or More	Other
	49 (1.0)	208 (4.3)	54 (1.1)	8 (0.2)	4428 (91.8)	31 (0.6)	47 (1.0)
Age Group –no. (%)	16–20	21–25	26–30	31–35	36–40	41–45	46–50
	86 (1.8)	178 (3.7)	192 (4.0)	213 (4.4)	341 (7.1)	375 (7.8)	415 (8.6)
	51–55	56–60	61–65	66–70	71–75	76–80	81+
	461 (9.6)	485 (10.1)	660 (13.7)	569 (11.8)	533 (11.0)	217 (4.5)	100 (1.9)
Phase – no. (%)	1a - HC	1b – 1st R	1b – 70+	1b – K12	1b - CC	1c - EW	1c - 16 + H
	1396 (28.9)	45 (0.9)	891 (18.5)	509 (10.4)	42 (0.9)	338 (7.0)	268 (5.6)
	2–60-69	3–15-59	Other				
	734 (15.2)	375 (7.8)	227 (4.8)				
Brand – no. (%)	P	M					
	4118 (85.3)	707 (14.7)					
Important Factor – no. (%)	Self	Others	Activities	Masks	Other		
	2980 (61.1)	1192 (24.7)	209 (4.3)	163 (3.4)	281 (5.8)		
Other Vaccines –no. (%)	n	Influenza	Tetanus	Pneumonia	Shingles		
	4786	4316 (90.2)	4222 (88.2)	2508 (52.6)	1887 (39.4)		
Prior COVID-19 – no. (%)	Yes	< 30 days	30–60 days	60–90 days	> 90 days		
	528 (10.8)	26 (4.9)	65 (12.3)	117 (22.2)	320 (60.6)		

The majority of respondents (85.3%) received the BNT162b2 vaccine. The percentages (Table 1) are reflective of the availability and distribution of vaccine types at the Med Center Health Vaccine Clinic from which the sample of respondents was drawn (M. Joyce, pers. comm.).

The majority of respondents (61.8%) cited the “ability to protect oneself from COVID-19” as the most important factor in taking the vaccine, while a substantial number (24.7%) cited “a desire to protect others” as their primary motivator (Table 1). A modest percentage (10.9%) reported having contracted COVID-19 previously. Of these, 60.6% were exposed > 90 days prior to receiving the vaccine (Table 1). The majority of respondents reported having previously received an influenza (90.2%) or tetanus (88.2%) vaccine, with smaller percentages having received vaccinations against pneumonia (52.6%) or shingles (39.4%) (Table 1).

### First dose side effects

The most commonly-reported side effects following the first dose of either the vaccine were arm soreness near the injection site, fatigue, muscle ache near the injection site, and headache; these were reported by greater than 25% of respondents (Table 2). Injection site redness, chills, and fever were reported in 10–25% of respondents. Other symptoms were reported by fewer than 10% (Table 2). Allergic reactions were the least-reported side effect, cited by only 2% of respondents (Table 2). No rare severe adverse effects – such as myocarditis, thrombosis, or hemorrhage – were reported.

**Table 2:** Distribution of self-reported side effect severities following the first dose of either the BNT162b2 (Brand = P, *n* = 4118) or mRNA-1273 (Brand = M, *n* = 707) vaccine, and comparisons between patients receiving the different vaccines. Physical side effects are ranked by incidence rate of symptoms, from highest to lowest. Side effects severities for physical symptoms were as follows: 1 = no symptoms; 2 = mild symptoms; 3 = moderate symptoms; 4 = severe symptoms; 5 = medical care required. Respondents indicate the need for medications following vaccination as follows: 1 = no; 2 = yes. The ratings scale for days of missed activities was as follows: 0, 1, 2, 3, and 4+ days. In every case except medications required, the mRNA-1273 vaccine elicited somewhat stronger responses; these differences were significant for 9 of 17 side effects

Side Effect	Brand	Side Effect Severity – no. (%)					Mean + SE	*p*-value
		1	2	3	4	5		
Arm soreness	P	1117 (27.1)	2134 (51.8)	760 (18.5)	104 (2.5)	3 (0.1)	1.97 + 0.01	< 0.001*
	M	131 (18.5)	367 (51.9)	187 (26.4)	22 (3.1)	0 (0.0)	2.14 + 0.03	
Fatigue	P	2581 (62.7)	931 (22.6)	452 (11.0)	53 (1.3)	1 (< 0.1)	1.56 + 0.01	< 0.001*
	M	401 (56.7)	173 (24.5)	80 (11.3)	53 (7.5)	0 (0.0)	1.70 + 0.04	
Muscle ache	P	2639 (64.1)	1058 (25.7)	321 (7.8)	97 (2.4)	3 (0.1)	1.49 + 0.01	< 0.001*
	M	392 (55.4)	211 (29.8)	83 (11.7)	21 (3.0)	0 (0.0)	1.62 + 0.03	
Headache	P	2985 (72.5)	768 (18.6)	272 (6.6)	92 (2.2)	1 (< 0.1)	1.39 + 0.01	0.006
	M	467 (66.1)	152 (21.5)	66 (9.3)	22 (3.1)	0 (0.0)	1.50 + 0.03	
Injection site redness	P	3487 (84.7)	552 (13.4)	57 (1.4)	19 (0.5)	3 (0.1)	1.18 + 0.01	< 0.001*
	M	496 (70.2)	166 (23.5)	33 (4.7)	12 (1.7)	0 (0.0)	1.38 + 0.02	
Chills	P	3542 (86.0)	340 (8.3)	175 (4.2)	59 (1.4)	2 (< 0.1)	1.21 + 0.01	< 0.001*
	M	570 (80.6)	65 (9.2)	45 (6.4)	27 (3.8)	0 (0.0)	1.33 + 0.03	
Fever	P	3698 (89.8)	265 (6.4)	107 (2.6)	48 (1.2)	0 (0.0)	1.15 + 0.01	< 0.001*
	M	598 (84.6)	59 (8.3)	28 (4.0)	21 (3.0)	1 (0.1)	1.26 + 0.03	
Nausea	P	3733 (90.7)	246 (6.0)	95 (2.3)	42 (1.0)	2 (< 0.1)	1.14 + 0.01	0.176
	M	627 (88.7)	49 (6.9)	17 (2.4)	14 (2.0)	0 (0.0)	1.18 + 0.02	
Injection site itching	P	3842 (93.3)	250 (6.1)	31 (0.8)	12 (0.3)	1 (< 0.1)	1.09 + 0.01	< 0.001*
	M	587 (83.0)	99 (14.0)	18 (2.5)	2 (0.3)	1 (0.1)	1.21 + 0.02	
Lymph node swelling	P	3900 (94.7)	166 (4.0)	41 (1.0)	9 (0.2)	2 (< 0.1)	1.07 + 0.01	0.072
	M	665 (94.1)	38 (5.4)	9 (1.3)	5 (0.7)	0 (0.0)	1.10 + 0.02	
Diarrhea	P	3901 (94.7)	156 (3.8)	40 (1.0)	19 (0.5)	2 (< 0.1)	1.07 + 0.01	0.698
	M	669 (94.6)	26 (3.7)	6 (0.8)	6 (0.8)	0 (0.0)	1.08 + 0.01	
Rash	P	3976 (96.6)	106 (2.6)	19 (0.5)	14 (0.3)	3 (0.1)	1.05 + 0.01	< 0.001*
	M	632 (89.4)	56 (7.9)	13 (1.8)	6 (0.8)	0 (0.0)	1.14 + 0.02	
General itching	P	3954 (96.0)	128 (3.1)	25 (0.6)	9 (0.2)	2 (< 0.1)	1.05 + 0.01	0.080
	M	663 (93.8)	36 (5.1)	5 (0.7)	2 (0.3)	1 (0.1)	1.08 + 0.01	
Vomiting	P	4020 (97.6)	57 (1.4)	18 (0.4)	20 (0.5)	3 (0.1)	1.04 + 0.01	0.445
	M	689 (97.5)	8 (1.1)	8 (1.1)	7 (1.0)	1 (0.1)	1.05 + 0.01	
Allergic reaction	P	4037 (98.0)	53 (1.3)	19 (0.5)	7 (0.2)	2 (< 0.1)	1.03 + 0.01	0.187
	M	684 (96.7)	14 (2.0)	6 (0.8)	3 (0.4)	0 (0.0)	1.05 + 0.01	
Impact	Brand	Additional Impacts – no. (%)					Mean + SE	*p*-value
		0	1	2	3	4		
Medication required	P	1421 (34.5)	2697 (66.5)				0.65 + 0.01	< 0.001*
	M	307 (43.4)	400 (56.6)				0.57 + 0.02	
Days missed activities	P	3714 (90.2)	249 (6.0)	92 (2.2)	36 (0.9)	27 (0.7)	0.16 + 0.01	0.664
	M	618 (87.4)	59 (8.3)	16 (2.3)	5 (0.7)	9 (1.3)	0.57 + 0.02	

*significant based on Bonferroni-adjusted criteria (p_crit_ = 0.05/17 = 0.003)

In all cases, side effects were generally quite mild. Arm soreness resulting from the first dose of mRNA-1273 had the highest mean severity (2.14 + 0.03), which was still in the mild category; all other side effects across both brands had mean severity values less than 2.00 (Table 2). The majority (64.2%) reported taking medications to alleviate their symptoms following the vaccine. The vast majority (89.8%) reported that the side effects did not cause them to miss work or other activities; of those who reported having missed activities, the majority indicated the number of days during which their activities were impacted was one (Table 2).

Reported side effects were more pronounced among respondents who received mRNA-1273. For nine of 17 side effects – arm soreness, fatigue, muscle ache, injection site redness, chills, fever, injection site itching, rash, and medications required – this difference was statistically-significant (Table 2).

### Second dose side effects

Second dose side effects were similar to those reported for first doses. Arm soreness, fatigue, muscle ache, and headache remained the most commonly-reported, followed by chills, fever, and injection site redness (Table 3). Allergic reactions were again quite rare, reported by only 2% of respondents. In most cases, second dose side effects were stronger than those reported for the first dose (Table 2, Table 3); the only exceptions were arm soreness (BNT162b2), injection site itching (both brands), and general itching (mRNA-1273). The magnitude of the differences was less than 5% in all cases. No rare severe adverse events were reported.

**Table 3:** Distribution of self-reported side effect severities following the second dose of either the BNT162b2 (Brand = P, *n* = 4118) or mRNA-1273 (Brand = M, *n* = 707) vaccine, and comparisons between patients receiving the different vaccines. Physical side effects are ranked by incidence rate of symptoms, from highest to lowest. Side effects severities for physical symptoms were as follows: 1 = no symptoms; 2 = mild symptoms; 3 = moderate symptoms; 4 = severe symptoms; 5 = medical care required. Respondents indicate the need for medications following vaccination as follows: 1 = no; 2 = yes. The ratings scale for days of missed activities was as follows: 0, 1, 2, 3, and 4+ days. In 15 of 17 cases, the mRNA-1273 vaccine elicited somewhat stronger responses; this difference was significant for 11 of those side effects. By contrast, lymph node swelling and medications required showed significantly lower mean responses in the mRNA-1273 group relative to the BNT162b2 group of respondents

Side Effect	Brand	Side Effect Severity – no. (%)					Mean + SE	*p*-value
		1	2	3	4	5		
Arm soreness	P	1321 (32.1)	2055 (49.9)	628 (15.3)	109 (2.6)	5 (0.1)	1.88 + 0.01	< 0.001*
	M	136 (19.2)	352 (49.8)	165 (23.3)	53 (7.5)	1 (0.1)	2.20 + 0.03	
Fatigue	P	2166 (52.6)	852 (20.7)	694 (16.9)	396 (9.6)	10 (0.2)	1.84 + 0.02	< 0.001*
	M	218 (30.8)	156 (22.1)	174 (24.6)	159 (22.5)	0 (0.0)	2.39 + 0.04	
Muscle ache	P	2467 (59.9)	933 (22.7)	498 (12.1)	212 (5.1)	8 (0.2)	1.63 + 0.01	< 0.001*
	M	257 (36.4)	199 (28.1)	152 (21.5)	99 (14.0)	0 (0.2)	2.13 + 0.04	
Headache	P	2680 (65.1)	770 (18.7)	475 (11.5)	190 (4.6)	3 (0.1)	1.56 + 0.01	< 0.001*
	M	318 (45.0)	182 (25.7)	128 (18.1)	79 (11.2)	0 (0.0)	1.95 + 0.04	
Chills	P	3133 (76.1)	407 (9.9)	343 (8.3)	228 (5.5)	7 (0.2)	1.44 + 0.01	< 0.001*
	M	358 (50.6)	108 (15.3)	121 (17.1)	120 (17.0)	0 (0.0)	2.00 + 0.04	
Fever	P	3412 (82.9)	293 (7.1)	244 (5.9)	165 (4.0)	4 (0.1)	1.31 + 0.01	< 0.001*
	M	424 (60.0)	93 (13.2)	98 (13.9)	90 (12.7)	2 (0.3)	1.80 + 0.04	
Injection site redness	P	3606 (87.6)	411 (10.0)	75 (1.8)	24 (0.6)	2 (< 0.1)	1.56 + 0.01	< 0.001*
	M	517 (73.1)	131 (18.5)	39 (5.5)	20 (2.8)	0 (0.0)	1.38 + 0.03	
Nausea	P	3603 (87.5)	276 (6.7)	145 (3.5)	90 (2.2)	4 (0.1)	1.21 + 0.01	< 0.001*
	M	539 (76.2)	70 (9.9)	64 (9.1)	33 (4.7)	1 (0.1)	1.43 + 0.03	
Lymph node swelling	P	3774 (91.6)	230 (5.6)	81 (2.0)	29 (0.7)	4 (0.1)	1.12 + 0.01	< 0.001*
	M	614 (86.8)	54 (7.6)	24 (3.4)	14 (2.0)	1 (0.1)	1.21 + 0.02	
Injection site itching	P	3846 (93.4)	230 (5.6)	28 (0.7)	14 (0.3)	0 (0.0)	1.08 + 0.01	< 0.001*
	M	605 (85.6)	79 (11.2)	17 (2.4)	6 (0.8)	0 (0.0)	1.19 + 0.02	
Diarrhea	P	3833 (93.1)	172 (4.2)	75 (1.8)	35 (0.8)	3 (0.1)	1.11 + 0.01	0.012
	M	632 (89.4)	46 (6.5)	20 (2.8)	9 (1.3)	0 (0.0)	1.16 + 0.02	
Rash	P	3972 (96.5)	103 (2.5)	27 (0.7)	14 (0.3)	2 (< 0.1)	1.05 + 0.01	< 0.001*
	M	639 (90.4)	46 (6.5)	15 (2.1)	7 (1.0)	0 (0.0)	1.14 + 0.02	
General itching	P	3970 (96.4)	113 (2.7)	27 (0.7)	8 (0.2)	0 (0.0)	1.05 + 0.01	0.191
	M	671 (94.9)	25 (3.5)	8 (1.1)	3 (0.4)	0 (0.0)	1.07 + 0.01	
Vomiting	P	3986 (96.8)	63 (1.5)	25 (0.6)	40 (1.0)	4 (0.1)	1.06 + 0.01	0.249
	M	676 (95.6)	15 (2.1)	9 (1.3)	6 (0.8)	1 (0.1)	1.08 + 0.02	
Allergic reaction	P	4029 (97.8)	51 (1.2)	22 (0.5)	10 (0.2)	6 (0.1)	1.04 + 0.01	0.520
	M	687 (97.2)	9 (1.3)	6 (0.8)	4 (0.6)	1 (0.1)	1.05 + 0.01	
Impact	Brand	Additional Impacts – no. (%)					Mean + SE	*p*-value
		0	1	2	3	4		
Medication required	P	2405 (58.4)	1713 (41.6)				1.42 + 0.01	< 0.001*
	M	280 (39.6)	427 (60.4)				1.60 + 0.02	
Missed activities	P	3281 (79.7)	525 (12.7)	189 (4.6)	62 (1.5)	61 (1.5)	0.32 + 0.01	< 0.001*
	M	428 (60.5)	189 (26.7)	62 (8.8)	20 (2.8)	8 (1.1)	0.57 + 0.03	

*significant based on Bonferroni-adjusted criteria (p_crit_ = 0.05/17 = 0.003)

Arm soreness had the highest mean severity following the second dose, though still in the mild category (Table 3). Only injection site itching resulting from mRNA-1273, and medications required following BNT162b2 showed less severe reactions following the second dose (Table 2, Table 3). The mean number of days in which activities were impacted following vaccination was zero to one (Table 3).

Second-dose side effects to mRNA-1273 were generally stronger than those to BNT162b2. Thirteen side effects showed significant differences in severity between brands. These included all 11 side effects that showed significant differences after the first dose, as well as additional side effects of headache, nausea, lymph node swelling, and missed activities (Table 3).

### Incidence rate ratios

Most side effects (12 of 17) tracked in our study were common to BNT162b2 and/or mRNA-1273 clinical trials. Incidence rate ratios (IRRs) indicated general consistency between our data and clinical trials. While the majority of IRRs comparisons were significantly different from zero, the magnitude of differences was of limited clinical significance in most cases. Only five comparisons were characterized by Cohen’s h values in the moderate (0.50 < h < 0.80) or large (0.80 < h < 1.0) range [21] (Table 4). Among BNT162b2 recipients, the use of pain medication was clinically greater than reported in the clinical trial (IRR = 2.69 + 0.12, *p* < 0.05, h = 0.85). Among mRNA-1273 recipients, injection site redness was clinically greater following the first dose compared to the clinical trial (IRR = 10.48 + 0.84, *P* < 0.05, h = 0.82), while fever was of moderate clinical significance following both first (IRR = 20.31 + 1.62, *p* < 0.05, h = 0.63) and second (IRR = 2.57 + 0.20, *p* < 0.05, h = 0.56) doses. Injection site redness was also of moderate clinical significance following the second dose of mRNA-1273 compared to the clinical trial (IRR = 3.14 + 0.26, *p* < 0.05, h = 0.50). Nevertheless, the severity in all cases was consistently in the mild to moderate range; fever following the second dose of mRNA-1273 was the only side effect in which the incidence of either a severe reaction or one requiring medical attention exceeded 5% (Tables 2, 3).

**Table 4:** Incidence rate ratios of side effects and impacts reported in this study vs. those reported in BNT162b2 and/or mRNA-1273 clinical trials. For first and second doses of each vaccine, incidence ratios (IR) and incidence rate ratios (IRR + 95% CI) are given for each side effect or impact common between our study and either of the clinical trials

Side Effect	BNT162b2				mRNA-1273			
	First Dose		Second Dose		First Dose		Second Dose	
	IR	IRR (+ CI)	IR	IRR (+ CI)	IR	IRR (+ CI)	IR	IRR (+ CI)
Arm soreness	62.9	0.81 (0.04)*	67.9	0.93 (0.05)*	81.5	0.97 (0.08)	80.8	0.91 (0.08)*
Fatigue	37.3	0.90 (0.04)*	47.4	0.85 (0.04)*	43.3	1.17 (0.09)	69.2	1.06 (0.09)
Muscle ache	35.9	1.99 (0.09)*	40.1	1.20 (0.06)*	44.6	1.97 (0.15)	63.6	1.10 (0.09)*
Headache	27.5	0.80 (0.04)*	34.9	0.76 (0.04)*	33.9	1.04 (0.08)	55.0	0.94 (0.07)
Chills	14.0	1.32 (0.06)*	23.9	0.81 (0.04)*	19.4	2.35 (0.19)	49.4	1.13 (0.08)*
Fever	10.2	1.88 (0.08)*	17.1	0.63 (0.03)*	15.4	20.31 (1.62)**	40.0	2.57 (0.20)**
Injection site redness	15.3	3.31 (0.15)*	12.4	1.92 (0.09)*	29.8	10.48 (0.84)***	26.9	3.14 (0.26)**
Nausea	9.3	–	12.5	–	11.3	1.36 (0.11)	23.8	1.26 (0.09)*
Lymph node swelling	5.3	–	8.4	–	5.9	0.58 (0.05)	13.2	0.94 (0.08)
Injection site itching	6.7	–	6.6	–	17.0	–	14.4	–
Diarrhea	5.3	0.54 (0.02)*	6.9	0.73 (0.03)*	5.4	–	10.6	–
Rash	3.4	–	3.5	–	10.6	–	9.6	–
General itching	4.0	–	3.6	–	6.2	–	5.1	–
Vomiting	2.4	2.65 (0.13)*	3.2	2.36 (0.11)*	2.5	–	4.4	–
Allergic reaction	2.0	–	2.2	–	3.3	–	2.8	–
Impact	BNT162b2				mRNA-1273			
	First Dose		Second Dose		First Dose		Second Dose	
	IR	IRR	IR	IRR	IR	IRR	IR	IRR
Medication required	65.5	2.69 (0.12)***	41.6	1.00 (0.05)*	56.6	–	60.4	–
Missed activities	9.8	–	20.3	–	12.6	–	39.5	–

*p < 0.05, Cohen’s h < 0.5

**p < 0.05, 0.5 < Cohen’s h < 0.80

***p < 0.05, Cohen’s h > 0.80

### Unsolicited adverse events

Respondents listed 531 “other” side effects across their first (*n* = 234, 4.8%) and second (*n* = 297, 6.2%) vaccine doses; however, more than 20% of these were identical in language to previously listed side effects. Most others listed included a general sense of feeling “unwell,” “bad,” or “flu-like.” Very few respondents (0.4%) listed potentially severe side effects such as seizures (*n* = 2), Bell’s palsy (*n* = 1), anaphylactic shock (*n* = 1), and heart arrhythmias (*n* = 2). Only two types of unsolicited adverse events were commonly reported: dizziness, including vertigo, brain fog and lightheadedness (*n* = 71); and changes in menstruation (*n* = 17).

### Gender and age differences

There was a significant difference in severity score as a function of gender, for both first (Kruskal-Wallis H = 95.33, df = 1, *p* < 0.001) and second (Kruskal-Wallis H = 208.08, df = 1, *p* < 0.001) doses; overall, females reported more severe reactions, though still in the generally mild category. There was a significant negative relationship between age and severity score for both first (b = − 0.019 + 0.001, *p* < 0.001) and second (b = − 0.037 + 0.002, *p* < 0.001) doses, though the relationships were relatively weak (*r*^2^ = 0.055 and *r*^2^ = 0.095, respectively).

### Comparisons between BNT162b2 and mRNA-1273 vaccines

**Fig. 1:**
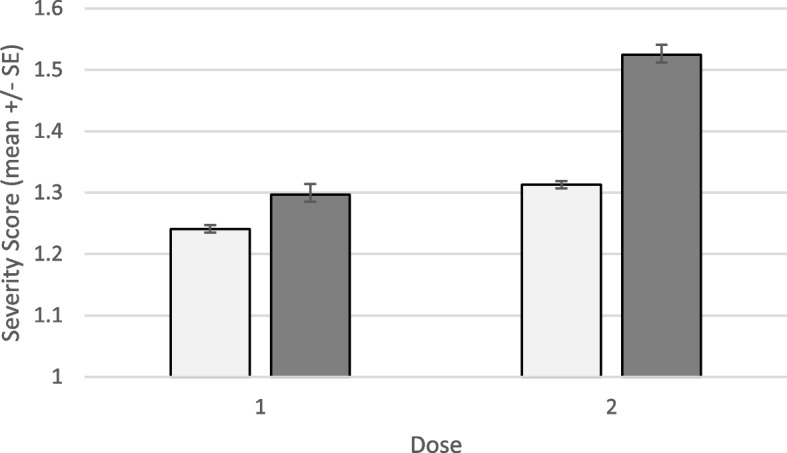
Mean severity score (+ SE) following first and second doses of BNT162b2 (light bars) or mRNA-1273 (dark bars)

Overall side effect severity scores were significantly higher among respondents who received the mRNA-1273 vaccine. This pattern was seen following both first (Kruskal-Wallis H = 54.22, df = 1, *p* < 0.001) and second (Kruskal-Wallis H = 220.66, df = 1, *p* < 0.001) doses (Fig. 1). Second-dose severity scores were significantly greater than first-dose severity scores for both BNT162b2 (Wilcoxon z = 4.42, *p* < 0.001) and mRNA-1273 (Wilcoxon z = 11.69, *p* < 0.001) (Fig. 1). Most interesting, the difference between first- and second-dose reactions was three times greater among mRNA-1273 as compared to BNT162b2 recipients (18% vs. 6%; Fig. 1).

### Reactions in COVID-19 positive versus COVID-19 Naïve respondents

**Fig. 2:**
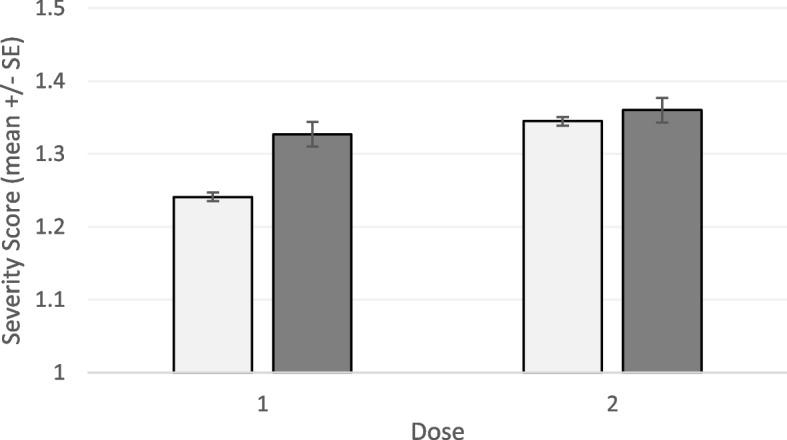
Mean severity score (+ SE) following first and second doses of the COVID-19 vaccine in COVID-19 naïve respondents (light bars) vs. those who reported a previous COVID-19 infection (dark bars). Data are pooled across vaccine types

Respondents who reported previously tested positive for COVID-19 showed significantly higher side effect severity scores following their first vaccine dose than did COVID-19 naïve respondents (Kruskal-Wallis H = 79.77, df = 1, *P* < 0.001; Fig. 2); first-dose reactions of COVID-19 positive respondents was on-par or slightly higher than the second-dose reactions seen among COVID-19 naïve respondents (Fig. 2). However, severity scores in COVID-19 positive respondents were only slightly higher than first-dose reactions in this same group (Kruskal-Wallis H = 6.31, df = 1, *p* < 0.01; Fig. 2).

### Correlations with responses to other vaccines

There was a positive correlation between severity scores calculated for first and second doses of both vaccines and respondents’ self-reported severity of reaction to previous influenza, pneumonia, tetanus, and/or shingles vaccines (Table 5). While the magnitude of these correlations was rather low (*r* < 0.23 in all cases), 11 of 16 comparisons were statistically significant (Table 5). The strongest correlations were seen between the first dose of BNT162b2 and other vaccines; in fact, first-dose correlations were higher than correlations observed from second-dose reactions (Table 5). By contrast, second-dose correlations of the mRNA-1273 vaccine with other vaccines were higher than first-dose correlations (Table 5).

**Table 5:** Comparisons of side effect severity scores from the BNT162b2 (Brand = P) or mRNA-1273 (Brand = M) and self-reported severity of side effects to four other vaccines

Variable	Brand	Other Vaccine	Sample Size (n)	Correlation (r)	*p*-value
First-dose severity score	P	Influenza	3658	0.233	< 0.001*
		Pneumonia	2105	0.170	< 0.001*
		Tetanus	3888	0.102	< 0.001*
		Shingles	1538	0.112	< 0.001*
	M	Influenza	647	0.153	< 0.001*
		Pneumonia	279	0.142	0.018
		Tetanus	611	0.048	0.238
		Shingles	189	0.034	0.642
Second-dose severity score	P	Influenza	3658	0.206	< 0.001*
		Pneumonia	2105	0.139	< 0.001*
		Tetanus	3888	0.085	< 0.001*
		Shingles	1538	0.073	0.003*
	M	Influenza	639	0.199	< 0.001*
		Pneumonia	274	0.187	0.002*
		Tetanus	603	0.058	0.160
		Shingles	186	0.050	0.499

*significant based on Bonferroni-adjusted criteria within families of tests (p_crit_ = 0.05/4 = 0.0125)

## Discussion

### Comparison with clinical trials and other data

Our findings derived from rollout of BNT162b2 and mRNA-1273 vaccines within a diverse, at-risk community align well with data from clinical trials. The most common side effects reported by our 4825 respondents – arm soreness, fatigue, muscle ache at the injection site, and headache – were the same as reported in both clinical trials [2, 3]. Chills, nausea, and fever were also commonly reported by both our respondents as well as those participating in clinical trials. The duration of side effects impacting regular activities among our group of respondents was generally zero to 2 days, also consistent with both trials [2, 3].

BNT162b2 [2] and mRNA-1273 [3] trials reported a higher incidence of both local and systemic side effects in younger participants compared to those in the older age categories. This is consistent with our finding of a significant negative relationship between side effect severity and age. Neither clinical trial compared incidence or severity of reactions between males and females, while our data indicated that females reported more severe impacts than did males, though generally in the mild range.

Our results are also consistent with previously published studies of real-world populations citing injection site pain or arm soreness as the highest reported side effect [4, 21, 37–39]. Fatigue and headache were also commonly reported, as were a range of other symptoms including swelling and tenderness at the injection site, chills and/or general malaise. Incidence rates of common symptoms were broadly consistent with what we observed, though there existed a fair amount of variation among studies; in particular, Menni et al. [21] reported lower incidence rates for all of these common side effects. Several studies mirrored our results and reported a higher incidence and/or severity among females as compared to males [21, 38] and younger vs. older recipients [21, 38]. Ripabelli et al. [38] attributed these trends to well-established differences in pharmacological responses between groups, including differences in cellular immune response between genders and the relative robustness of the immune system in younger versus older individuals.

As in previous studies [4, 21, 37–39], we found evidence for increased side effects following the second dose of the vaccine. Our data also support prior data indicating stronger reactions to mRNA-1273 than to BNT162b2 [2, 3, 39, 40], particularly following the second dose. This difference may reflect the higher dose amount of mRNA-1273 (100 vs. 30 micrograms), and longer inter-dose interval (28 vs. 21 days). While these differences may account for the consistently higher side effect severity associated with mRNA-1273, neither fully explains the fourfold greater difference in severity between first and second doses of mRNA-1273. In fact, the one-week greater interval between doses in the mRNA-1273 series – assuming a linear relationship between inter-dose interval and side effect severity – would be expected to result in only an 8% greater difference, vs. the 18% observed.

Other adverse side effects, beyond those explored in trails, have been noted in response to the vaccine, and our incidence rates of such effects closely mirror other similar investigations [38]. Serious side effects of note include neurological symptoms [41], anaphylaxis [42, 43], and myocarditis [44, 45]. Our respondents listed very few if any of these types of events. However, the prevalence of dizziness and changes in menstruation may warrant further exploration. Dizziness, light-headiness and brain fog are similar to reported symptoms of COVID-19 infection [46]. The basis for our respondents’ symptoms are unclear, but other studies have found dizziness to be a common neurological side effect of the vaccine [47]. Ripabelli et al. [38] suggested this might reflect anxiety-related symptoms associated with the vaccination process. Menstruation-related symptoms have also been reported elsewhere [48], and a National Institutes of Health funding initiative was established to investigate these effects [49].

These findings support the conclusion that side effects experienced by individuals in a diverse population, characterized by a range of comorbidities, are likely to be similar in pattern and severity to what has been described from clinical trials of healthy individuals. Practitioners, health departments, and other entities can thus have confidence in promoting both BNT162b2 and mRNA-1273 as safe and minimally-disruptive of normal activities.

### Responses in individuals with prior history of COVID-19 infection

Individuals in our sample with a previously-documented COVID-19 infection reported a higher level of side effect severity than did COVID-19 naïve respondents, across both vaccine types. However, this increased severity was only significant following the first dose. Moreover, first-dose reactions in respondents previously infected with COVID-19 mirrored second-dose reactions in COVID-19 naïve respondents. Mathioudakis et al. [4] were among the first the show the link between prior COVID-19 diagnosis an increased risk of incidence or severity of vaccine side effects. A similar finding was reported by Ebinger et al. [50], who compared 35 patients with prior COVID-19 infection to an additional 528 COVID-19 naïve individuals. While smaller in scope than our study, these authors used antibodies to confirm prior COVID-19 infection.

Menni et al. [21] studied more than 2000 individuals previously diagnosed with COVID-19 who received the BNT162b2 vaccine. They found local side effects were one- to two-times more common, and systematic side effects two- to nine-times more common in such individuals [21].

Applying a multivariable logistic regression approach to a same of over 19,000 individuals, Beatty et al. [39] found that prior COVID-19 infection led to a significantly higher odds ratio of adverse effects following COVD-19 vaccination, second only to vaccine dose in terms of magnitude. Jeskowiak et al., [37] found previously diagnosed individuals had stronger adverse effects after the first dose of the vaccine, which they postulated could be due to a weakened antibody-dependent enhancement, whereas COVID-19 naïve respondents experienced stronger side effects after the second dose. Taken together, these findings suggest that prior COVID-19 infection may effectively serve as a priming dose for the immune response (analogous to the first vaccine dose in COVID-19 naïve individuals). The similarity of second-dose responses in both COVID naïve respondents and those with prior COVID-19 infection in our study further suggests responses to booster doses might be expected to be on par with individuals’ second doses. Such a pattern, if confirmed by subsequent studies, could encourage individuals to take booster doses when recommended.

### Comparisons with other vaccines

There was a positive correlation between severity of side effects to either COVID-19 vaccine and individuals’ reported responses to other vaccines. Individuals who reported a higher incidence or severity of side effects to the BNT162b2 vaccine also tended to report a higher level of overall severity of responses to influenza, pneumonia, tetanus, and shingles vaccines; these correlations were significant across both first and second doses. With mRNA-1273, we only observed significant correlations between the influenza vaccine and either dose of the COVID-19 vaccine, and between the pneumonia vaccine and the second dose of the mRNA-1273 series. In all cases, however, the correlations were low. A similar positive association between adverse events resulting from the second dose of the COVID-19 vaccine and prior vaccinations was reported by Ripabelli et al. [38]. These data suggest that individuals might expect to respond to COVID-19 vaccination similarly to their response to other vaccines. Conversely, Beatty et al. [39] found that receiving an influenza shot during the prior year was associated with lower odds of severe a response to COVID-19 vaccination. While the relationship between COVID-19 and other vaccines remains somewhat unresolved, such data could be useful to individuals in making the decision as to whether and how to schedule their COVID-19 vaccination.

### The utility of community-based vaccination data to address vaccine hesitancy

Measuring or affecting vaccine hesitancy was not a primary objective of this research. However, our results have been used in local community-led vaccination efforts, and it is prudent to discuss the potential of this study, and those like it, in persuading the vaccine hesitant. This is particularly important in regions like Kentucky which are characterized by a high level of comorbidities that increase susceptibility to severe COVID-19 [22, 24] and political and social-economic factors that increase the rate of vaccine resistance [51, 52] and continue to this day.

Research suggests that vaccine holdouts may be reachable with the right information provided by trusted sources [9, 10, 53, 54], (Vlasceanu M, Coman A: The impact of information sources on Covid-19 knowledge accumulation and vaccination intention, forthcoming). Use of local messaging [55, 56], and social norms [57] are thought to increase trust and serve as effective motivators for behavioral change. Message source is vitally important in persuasion [55, 58] as shared values between the messenger and the audience affect message acceptance [59], especially among conservatives and moderates [60]. This “local source” phenomenon is related to the use of normative behavior, or social norms [61], which are included in various models and theories of health-related behavior change [57, 61, 62]. Research has also demonstrated that behavior change is most likely when information comes of an individual’s own social network [63–65], and that social norms and circles can influence health behaviors and attitudes toward vaccination uptake [66, 67] especially via social media [18]. Additionally, interventions targeted at individual healthcare teams and their patients can optimize efforts to address vaccine hesitancy [68].

Given these considerations, local research projects such as ours have increased credibility and potential to persuade at the community level. Our results were reported by local news outlets and on social media. Study participants were our own community members, which increases the normative value of getting vaccinated. Further, the large number of participants means individuals from many different social circles in our community were likely engaged.

The current vaccine-hesitant moment in which we find ourselves will continue to impact the public health sector in the context of COVID-19 and beyond. The return of measles to the world-stage is a cautionary tale. Eradicated from the U.S. in 2000, there were 1200 reported cases in 2019, while Europe saw more the 90,000 in the first half of the year. Increases across the African continent have also been reported, and in most cases, these increases are tied to precipitous declines in vaccination rates [31]. Similar trends have also been noted for the seasonal flu and HPV [17]. Additionally, COVID-19 is certain not be the last outbreak to require a rapid vaccine rollout, the cited cause for increased misinformation [17]. In fact, the current outbreak of monkeypox recently prompted the U.S. FDA to authorize the same “emergency use authorization” for the JYNNEOS vaccine (Modified Vaccinia Ankara, MVA) against monkeypox [68].

Given these disturbing anti-vaccine trends and the inevitability of novel and resurgent viruses, it is prudent to identify strategies targeting vaccine hesitancy and combatting misinformation [69] at the community level. This study was quick and inexpensive; it could be easily recreated to measure the side-effects of recurring (e.g., seasonal flu), childhood (e.g., MMR and HPV) and novel vaccine programs (e.g., monkeypox). Use of such locally sourced data in vaccine campaigns, even outside of the COVID-19 pandemic, and especially through social media [18], may be a strategy worth pursuing.

### Significance and limitations

We acknowledge several limitations with our study. As with any survey, results are dependent upon self-reporting of both side effects and severity level. This limitation was most evident with regards to unsolicited side effects, as respondents commonly listed side effects that were included in Likert scale questions. Since many respondents were healthcare workers, there may have been a bias towards over-reporting of side effects, as this population was attuned to side effects that had been reported in clinical trials. Additionally, it had been several months since some respondents received their COVID-19 vaccine, and they may not have accurately remembered their side effects and/or severity, potentially leading to overstating or understating symptoms. This same recall bias may have influenced respondents’ assessment of their responses to other vaccinations [70] and has been documented in other analysis of cognitive bias and vaccine-induced adverse events [71]. Some respondents may not have known they were previously COVID-19 positive, especially if they had been asymptomatic or had very mild symptoms; however, this represents a conservative error with respect to our finding of significant differences in response of prior COVID-19 positive vs. COVID-19 naïve individuals. We also did not ask respondents about any comorbid conditions, though regional demographics suggest that people with comorbid conditions were included in the sample.

## Conclusions

Our study provides an important contribution to the literature base on reactions to COVID-19 vaccination. Our data comprise nearly 5000 recipients from a population characterized by high levels of comorbidities, providing broad-scale estimates of the nature and severity of reactions one might expect within a clinically diverse community. Moreover, our study provides direct comparison of side effects between BNT162b2 and mRNA-1273 vaccines, which have to date not been widely considered. Our study adds to existing literature suggesting a relationship between prior COVID-19 infection and severity of response to subsequent vaccination, as well as association between reactions to the COVID-19 vaccine and other vaccinations. Finally, our data provide a context for addressing ongoing vaccine hesitancy in communities such as ours, where locally-generated data and communication may be more influential than national trends and statistics in convincing individuals to become vaccinated and/or take advantage of variant-specific boosters, as new vaccines for other infectious agents become available and receive similar testing and rapid authorization, and to combat vaccine hesitancy more widely, in other arenas (e.g., annual vaccines, childhood vaccines).

## Supplementary Information

**Additional file 1:.** Survey tool.

## Data Availability

The survey questionnaire used in this study was created by the authors of this publication, the research team and is attached as supplementary information. The survey questionnaire is attached as a supplement and the data used in this study are available from the corresponding author upon request.

## References

[1] Hervé C, Laupèze B, Del Giudice G, Didierlaurent AM, Tavares Da Silva F (2019). The how’s and what’s of vaccine reactogenicity. NPJ Vacc.

[2] Center for Disease Control and Prevention (2020). Local reactions, systemic reactions, adverse events, and serious adverse events: Pfizer-BioNTech COVID-19 vaccine.

[3] Center for Disease Control and Prevention. (2020). The Moderna COVID-19 vaccine’s local reactions, systemic reactions, adverse events, and serious adverse events. https://www.cdc.gov/vaccines/covid-19/info-by-product/moderna/reactogenicity.html.

[4] Mathioudakis AG, Ghrew M, Ustianowski A, Ahmad S, Borrow R, Papavasileiou LP (2021). Self-reported real-world safety and reactogenicity of COVID-19 vaccines: a vaccine recipient survey. Life.

[5] Graham BS (2020). Rapid COVID-19 vaccine development. Science.

[6] Mellet J, Pepper MS (2021). A COVID-19 vaccine: big strides come with big challenges. Vaccines.

[7] U.S. Department of Health and Human Services (2020). Experimental coronavirus vaccine is safe and produces immune response.

[8] Freeman D, Loe B, Chadwick A, Vaccari C, Waite F, Rosebrock L, et al. COVID-19 vaccine hesitancy in the UK: the Oxford coronavirus explanations, attitudes, and narratives survey (oceans) II. Psychol Med. 2020:1–15. 10.1017/S0033291720005188 PMC7804077 33305716

[9] King WC, Rubenstein M, Reinhart A, Mejia R (2021). COVID-19 vaccine hesitancy January-May 2021 among 18-64 year old US adults by employment and occupation. Prev Med Rep.

[10] King, W.C., Rubenstein, M., Reinhart, A., & Mejia, R.J. (2021). Time trends, factors associated with, and reasons for COVID-19 vaccine hesitancy in US adults: January-May 2021. medRxiv, 07.20.21260795. 10.1371/journal.pone.0260731 PMC8691631 34932583

[11] de Albuquerque Veloso Machado M, Roberts B, Wong BLH, van Kessel R, Mossialos E (2021). The relationship between the COVID-19 pandemic and vaccine hesitancy: a scoping review of literature until august 2021. Front Public Health.

[12] Centers for Disease Control and Prevention (2022). COVID Data Tracker.

[13] Limbu YB, Gautam RK, Pham L (2022). The health belief model applied to COVID-19 vaccine hesitancy: a systematic review. Vaccines.

[14] Wei WE, Tan WK, Cook AR, Hsu LY, Teo YY, Lee VJM (2021). Living with COVID-19: the road ahead. Ann Acad Med Singap.

[15] World Health Organization. 2019. Ten threats to global health in 2019. Available online: https://www.who.int/news-room/spotlight/ ten-threats-to-global-health-in-2019

[16] Gunasekera L, Wijeratne T (2021). Vaccine hesttancy during the coronavirus pandemic – lessons from polio. Life.

[17] Larson HJ, Gakidou E, Murray CJL (2022). The vaccine-hesitant moment. N Engl J Med.

[18] Clark SE, Bledsoe MC, Harrison CJ (2022). The role of social media in promoting vaccine hesitancy. Curr Opin Pediatr.

[19] World Health Organization (2022). WHO coronavirus disease (COVID-19) dashboard.

[20] Olivera Mesa D, Hogan AB, Watson OJ, Charles GD, Hauck K, Ghani AC, et al. Modelling the impact of vaccine hesitancy in prolonging the need for non-pharmaceutical interventions to control the COVID-19 pandemic. Commun Med. 2:14. 10.1038/s43856-022-00075-x. 10.1038/s43856-022-00075-x PMC9053271 35603311

[21] Menni C, Klaser K, May A, Polidori L, Capdevila J, Louca P (2021). Vaccine side-effects and SARS-CoV-2 infection after vaccination in users of the COVID symptom study app in the UK: a prospective observational study. Lancet Infect Dis.

[22] Alcendor DJ (2021). Targeting COVID vaccine hesitancy in rural communities in Tennessee: implications for extending the COVID-19 pandemic in the south. Vaccines.

[23] America’s Health Rankings (2021). Annual report for Kentucky, United Health Foundation.

[24] Fang L, Karakiulakis G, Roth M (2020). Are patients with hypertension and diabetes mellitus at increased risk for COVID-19 infection?. Lancet Respir Med.

[25] Webb Hooper M, Napoles AM, Perez-Stable EJ (2020). COVID-19 and racial/ethnic disparities. J Am Med Assoc.

[26] Centers for Disease Control and Prevention (2021). People with certain medical conditions.

[27] Centers for Disease Control and Prevention (2021). COVID data tracker.

[28] Ballotopedia (2021). Presidential voting trends in Kentucky.

[29] Mathieu E, Ritchie H, Ortiz-Ospina E, Roser M, Hasell J, Appel C (2021). A global database of COVID-19 vaccinations. Nat Hum Behav.

[30] Halstead IN, McKay RT, Lewis GJ (2022). COVID-19 and seasonal flu vaccination hesitancy: links to personality and general intelligence in a large, UK cohort. Vaccine.

[31] Hotez P, Nuzhath T, Colwell B (2020). Combating vaccine hesitancy and other 21st century social determinants in the global fight against measles. Curr Opin Virol.

[32] Kentucky Department of Public Health (2021). Kentucky Vaccination Plan.

[33] Dillman DA, Smyth J, Christian L (2009). Internet, mail, and mixed-mode surveys: the tailored design method.

[34] Millar M, Dillman DA (2011). Improving response to web and mixed-mode surveys. Public Opinion Quarterly.

[35] Cohen J (1988). Statistical power analysis for the behavioral sciences.

[36] Bowling Green Chamber of Commerce (2021). Demographics.

[37] Jęśkowiak I, Wiatrak B, Grosman-Dziewiszek P, Szeląg A (2021). The incidence and severity of post-vaccination reactions after vaccination against COVID-19. Vaccines.

[38] Ripabelli G, Tamburro M, Buccieri N, Adesso C, Caggiano V, Cannizzaro F, et al. Active surveillance of adverse events in Hhalthcare workers recipients after vaccination with COVID-19 BNT162b2 vaccine (Pfizer-BioNTech, Comirnaty): a cross-sectional study. J Community Health. 2021;2021. 10.1007/s10900-021-01039-3 PMC8501918 34628568

[39] Beatty AL, Peyser ND, Butcher XE, Cocohoba JM, Lin F, Oglin JE (2021). Analysis of COVID-19 vaccine type and adverse effects following vaccination. JAMA Netw Open.

[40] Baden LR, El Sahly HM, Essink B, Kotloff K, Frey S, Novak R (2021). Efficacy and safety of the mRNA-1273mRNA-1273 SARS-CoV-2 vaccine. N Engl J Med.

[41] Waheed S, Bayas A, Hindi F, Rizvi Z, Espinosa PS (2021). Neurological complications of COVID- 19: Guillain-Barre syndrome following Pfizer COVID-19 vaccine. Cureus.

[42] Centers for Disease Control and Prevention (2020). Allergic reactions including anaphylaxis after receipt of the first dose of Pfizer-BioNTech COVID-19 vaccine — United States, December 14-23, 2020.

[43] Centers for Disease Control and Prevention (2021). Allergic reactions including anaphylaxis after receipt of the first dose of Moderna COVID-19 vaccine — United States, December 14, 2020 - -January 10, 2021.

[44] Aye YN, Mai AS, Zhang A, Lim OZH, Lin N, Ng CH, et al. Acute myocardial infarction and myocarditis following COVID-19 vaccination. QJM. 2021; hcab252, Advance online publication. 10.1093/qjmed/hcab252 PMC8522388 34586408

[45] Kim HW, Jenista ER, Wendell DC, Azevedo CF, Campbell MC, Darty SN (2021). Patients with acute myocarditis following mRNA COVID-19 vaccination. J Am Med Assoc Cardiol.

[46] Boldrini M, Canoll PD, Klein RS (2021). How COVID-19 affects the brain. J Am Med Assoc Psychiatry.

[47] Goss AL, Samudralwar RD, Das RR, Nath A (2021). ANA investigates: neurological complications of COVID-19 vaccines. Ann Neurol.

[48] Male V (2021). Menstrual changes after covid-19 vaccination. BMJ.

[49] National Institutes of Health (2021). RFP. Funds studies to assess potential effects of COVID-19 vaccination on menstruation.

[50] Ebinger JE, Fert-Bober J, Printsev I, Wu M, Sun N, Prostko (2021). Antibody responses to the BNT162b2 mRNA vaccine in individuals previously infected with SARS-CoV-2. Nat Med.

[51] Kates J, Tolbert J, Orgera K (2021). The red/blue divide in COVID-19 vaccination rates.

[52] Pew Research Center (2021). Majority in U.S. says public health benefits of COVID-19 restrictions worth the costs, even as large shares also see downsides.

[53] Foundation for a Healthy Kentucky (2021). Half of vaccine-hesitant KY adults open to changing mind on COVID-19 vaccine with more information.

[54] American Psychological Association (2021). Building vaccine confidence through community engagement.

[55] McGuire W, Gardner L, Aaronson E (1985). Attitudes and attitude change. Handbook of social psychology.

[56] Malik AA, McFadden SM, Elharake J, Omer SB (2020). Determinants of COVID-19 vaccine acceptance in the US. EClinical Medicine.

[57] Ajzen I (1991). The theory of planned behavior. Organ Behav Hum Decis Process.

[58] Wilson EJ, Sherrell DL (1993). Sources effects in communication and persuastion research: a meta-analysis of effect size. J Acad Mark Sci.

[59] Cvetkovich G, Cvetkovich G, Lofstedt R (1999). The attribution of social trust. Social trust and the management of risk.

[60] Bloodhart B, Maibach E, Myers T, Zhao X (2015). Local climate experts: the influence of local TV weather information on climate change perceptions. PLoS One.

[61] Cialdini RB, Trost MR, Gilbert DT, Fiske ST, Lindzey G (1998). Social influence, social norms, conformity and compliance. The handbook of social psychology.

[62] Dempsey RC, McAlaney J, Bewick BM (2018). A critical appraisal of the social norms approach as an interventional strategy for health-related behavior and attitude change. Front Psychol.

[63] Brewer NT (2021). What works to increase vaccination uptake. Acad Pediatr.

[64] Centola D (2010). The spread of behavior in an online social network experiment. Science.

[65] Centola D (2015). The social origins of networks and diffusion. Am J Sociol.

[66] Bruine de Bruin W, Parker AM, Galesic M, Vardavas R (2019). Reports of social circles’ and own vaccination behavior: a national longitudinal survey. Health Psychol.

[67] Moore R, Purvis RS, Hallgren E, Willis DE, Hall S, Reece S, et al. Motivations to vaccinate among hesitant adopters of the COVID-19 vaccine. J Community Health. 2021;2021. 10.1007/s10900-021-01037-5 PMC8536476 34687388

[68] Food and Drug Administration News Release. (2022). Monkeypox update: FDA authorizes emergency use of JYNNEOS vaccine to increase vaccine supply. https://www.fda.gov/news-events/press-announcements/monkeypox-update-fda-authorizes-emergency-use-jynneos-vaccine-increase-vaccine-supply.

[69] Lee, S.K., Sun, J., Jang, S. et al. Misinformation of COVID-19 vaccines and vaccine hesitancy. Sci Rep, 12:13681. 10.1038/s41598-022-17430-6. 10.1038/s41598-022-17430-6 PMC9366757 35953500

[70] Van Heiden S, Carrico R, Wiemken TL, Alexander R, McLaughlin JM, Jiang Q (2017). Level of recall bias regarding pneumococcal vaccination history among adults hospitalized with community-acquired pneumonia: results from the University of Louisville Pneumonia Study. Univ Louisville J Respir Infect.

[71] Azarpanah H, Farhadloo M, Vahidov R, Pilote L (2021). Vaccine hesitancy: evidence from an adverse events following immunization database, and the role of cognitive biases. BMC Public Health.

