# Supplementary material for: From trial to practice: incidence and severity of COVID-19 vaccine side effects in a medically at-risk and vaccine-hesitant community
Source: BMC Public Health. 2022 Dec 14;22:2351. doi: 10.1186/s12889-022-14824-z (PMC9750730; doi:10.1186/s12889-022-14824-z)
Supplement: Supplementary file 1 — Additional file 1:. Survey tool. [file 12889_2022_14824_MOESM1_ESM.docx]

Supplement

Survey Tool

This survey was created by the research team that has authored this publication.

**Start of Block: CONSENT**

Q27 Informed Consent: This project is in partnership with Med Center Health and Western Kentucky University. We are asking for your participation in our research study entitled, “Response to the COVID-19 Vaccine”. The purpose of this survey is to gain better insight into the responses or side effects that individuals may have experienced from the COVID-19 vaccine. The accompanying survey asks questions about potential side effects specific to the different types of COVID-19 vaccines. You are being asked to participate in this study if you received a COVID-19 vaccine sometime since December 14, 2020. This survey is anonymous. It will not ask for any protected health information nor will any protected health information be associated with your answer choices. Your answer choices to the survey questions will be analyzed statistically as part of a larger group and will not be reported for any single individual. The information gathered from this survey may be used in a scientific and/or medical journal publication. This journal may be online, free, and accessible worldwide.

Similarly, this information may be used for other materials, such as presentations at conferences, seminars, etc. Would you like to participate? The purpose of this form is to obtain consent to use information provided by you through the following survey. As a volunteer in this study, you may leave the survey at any point without finishing all questions. However, incomplete surveys will not be included in the data set. By clicking the “I accept these terms” button, you indicate that you have read and agree to the terms of this patient consent form and will be taken to the first question of the survey. This survey is designed to work well on your mobile device. However, depending on your screen size, you may need to "scroll down" to complete some sections. This survey will remain open through Monday, June 14, 2021. You will receive one reminder text asking you to participate. The study has been approved by the Med Center Health IRB #1 as 21-06-04-Joyce-RespC19Vacc. If you have any questions about this study, please contact Melinda Joyce, Pharm.D., at 270-745-1599 or [joycmc@mchealth.net.](mailto:joycmc@mchealth.net) Thank you for your time.

Q28 After reading the consent form, do you agree to participate in this study?

o Yes, I accept these terms and would like to participate. (1)

o No. (2)

**End of Block: CONSENT**

**Start of Block: Enter to Win**

Q32 As a thank you for participating in our study, we would like to enter you in our drawing for five $50 Amazon gift cards on June 20, 2021. If you would like to be entered, please provide

your email below. This information is kept separate from any other data or answers provided, and will not be used in any identifying way, nor for further contact.

**End of Block: Enter to Win**

**Start of Block: Age**

Q2 What is your age?

o 16-20 (1)

o 21-25 (2)

o 26-30 (3)

o 31-35 (4)

o 36-40 (5)

o 41-45 (6)

o 46-50 (7)

o 51-55 (8)

o 56-60 (9)

o 61-65 (10)

o 66-70 (11)

o 71-75 (12)

o 76-80 (13)

o 81-85 (14)

o 86-90 (15)

o 91-95 (16)

o 96-100 (17)

o 101 or older (18)

**End of Block: Age**

**Start of Block: Gender**

Q1 What is your gender?

o Male (1)

o Female (2)

o Non-binary / third gender (3)

o Prefer not to say (4)

**End of Block: Gender**

**Start of Block: Race/Ethnicity**

Q4 Which best describes you? Please select one.

o Asian or Pacific Islander (1)

o Black or African American (2)

o Hispanic or Latino (3)

o Native American or Alaskan Native (4)

o White or Caucasian (5)

o Multiracial or Biracial (6)

o Other (7)

o Prefer not to say (8)

**End of Block: Race/Ethnicity**

**Start of Block: Vaccinated?**

Q5 Have you received a COVID-19 vaccine?

o Yes (1)

o No (2)

**End of Block: Vaccinated?**

**Start of Block: No Vaccine**

Q29 Why have you not received the COVID-19 vaccine?

o Scheduling problems (1)

o It's not my turn yet (2)

o Technical difficulties (3)

o Afraid of side effects (4)

o Not convinced it is safe (5)

o Not convinced it is effective (6)

o Other (7)

**End of Block: No Vaccine**

**Start of Block: Vaccine WHY**

Q28 What was the most important factor in your decision to receive the COVID-19 vaccine?

o Protection from getting sick (1)

o Preventing infection to others (2)

o To participate in more activities outside the home (3)

o To minimize the use of masks (4)

o Other (5)

**End of Block: Vaccine WHY**

**Start of Block: Vaccine Phase**

Q6 Do you know under which designation or phase (1, 2, or 3) you received the vaccine?

o Healthcare worker (Phase 1a) (1)

o First responder (Phase 1b) (2)

o School personnel (Phase 1b) (3)

o Child Care personnel (Phase 1b) (4)

o Senior (age 70 and older) (Phase 1b) (5)

o Essential Worker (Phase 1c) (6)

o Underlying health concerns (Phase 1c) (9)

o Between 60 and 69 years of age (Phase 2) (10)

o Between 16 and 59 years of age (Phase 3) (11)

o I might have been in the wrong phase (8)

o I'm not sure (7)

**End of Block: Vaccine Phase**

**Start of Block: Brand**

Q7 Which brand of vaccine did you receive?

o Moderna (1)

o Pfizer (2)

o Johnson & Johnson (also referred to as Janssen) (4)

o I'm not sure (3)

**End of Block: Brand**

**Start of Block: J&J Vaccine Rxn**

Q18 Following your COVID-19 vaccine, how would you rate the severity of these side effects on the following scale?

| None: No symptoms (1) | | Mild: Did not interfere with activities (2) | Moderate: Interfered with some activities (3) | Severe: Prevented regular daily activity (4) | Medical Care Required: ER, doctor, or hospital (5) |
| --- | --- | --- | --- | --- | --- |
| Arm soreness (1) | o | o | o | o | o |
| Muscle pain/ joint pain (25) | o | o | o | o | o |
| Headache (26) | o | o | o | o | o |
| Fever (99+ degrees ) (27) | o | o | o | o | o |
| Chills(28) | o | o | o | o | o |
| Nausea (29) | o | o | o | o | o |
| Vomiting (30) | o | o | o | o | o |
| Diarrhea (31) | o | o | o | o | o |
| Fatigue (32) | o | o | o | o | o |
| Redness around injection site (33) | o | o | o | o | o |
| Skin Rash around injection site (34) | o | o | o | o | o |
| Itching around injection site only (35) | o | o | o | o | o |

| Generalized itching (36) | o | o | o | o | o |
| --- | --- | --- | --- | --- | --- |
| Swollen lymph glands (37) | o | o | o | o | o |
| Allergic reaction (last in this set, proceed after answering) (38) | o | o | o | o | o |

**End of Block: J&J Vaccine Rxn**

**Start of Block: Other JJ**

Q33 Did you experience any other symptoms not already listed?

o Yes I did. (Please describe below.) (2)

o No (1)

**End of Block: Other JJ**

**Start of Block: J&J Additional Qs**

Q19 Did you take any medications (either acetaminophen or a non-steroidal anti-inflammatory agent, such as ibuprofen) to treat the side effects of your COVID-19 vaccine?

o Yes (2)

o No (1)

Q20 How many days of work or regular activities did you miss as a result of the side effects of your COVID-19 vaccine?

o zero days (1)

o 1 day (2)

o 2 days (3)

o 3 days (4)

o 4 days or more (5)

**End of Block: J&J Additional Qs**

**Start of Block: Both Doses? (Moderna/Pfizer)**

Q8 Have you received BOTH doses of the vaccine?

o Yes (1)

o No (2)

**End of Block: Both Doses? (Moderna/Pfizer)**

**Start of Block: 1st Vaccine Rxn**

Q31 Following **your first** COVID-19 vaccine, how would you rate the severity of these side effects on the following scale?

| None: No symptoms (1) | | Mild: Did not interfere with activities (2) | Moderate: Interfered with some activities (3) | Severe: Prevented regular daily activity (4) | Medical Care Required: ER, doctor, or hospital (5) |
| --- | --- | --- | --- | --- | --- |
| Arm soreness (1) | o | o | o | o | o |
| Muscle pain/ joint pain (25) | o | o | o | o | o |
| Headache (26) | o | o | o | o | o |
| Fever (99+ degrees ) (27) | o | o | o | o | o |
| Chills (28) | o | o | o | o | o |
| Nausea (29) | o | o | o | o | o |
| Vomiting (30) | o | o | o | o | o |
| Diarrhea (31) | o | o | o | o | o |
| Fatigue (32) | o | o | o | o | o |
| Redness around injection site (33) | o | o | o | o | o |
| Skin Rash around injection site (34) | o | o | o | o | o |
| Itching around injection site only (35) | o | o | o | o | o |

| Generalized itching (36) | o | o | o | o | o |
| --- | --- | --- | --- | --- | --- |
| Swollen lymph glands (37) | o | o | o | o | o |
| Allergic reaction (last in this set, proceed after answering) (38) | o | o | o | o | o |

**End of Block: 1st Vaccine Rxn**

**Start of Block: Other 1st**

Q34 Did you experience any other symptoms not already listed with **your first** Covid-19 vaccine?

o Yes I did. (Please describe below.) (2)

o No (1)

**End of Block: Other 1st**

**Start of Block: 1st Vaccine Additional Qs**

Q24 Did you take any medications (either acetaminophen or a non-steroidal anti-inflammatory agent, such as ibuprofen) to treat the side effects of **your first** COVID-19 vaccine?

o Yes (1)

o No (2)

Q25 How many days of work or regular activities did you miss as a result of the side effects of

**your first** COVID-19 vaccine?

o zero days (1)

o 1 day (2)

o 2 days (3)

o 3 days (4)

o 4 days or more (5)

**End of Block: 1st Vaccine Additional Qs**

**Start of Block: Why No 2nd Dose**

Q10 Why have you NOT RECIEVED **your second** COVID-19 vaccine?

o My second appointment is not until a later date (1)

o I did not want to get second dose due to side effects (2)

o I could not get second dose due to testing positive for COVID-19 (3)

o I forgot my appointment (4)

o Other (5)

**End of Block: Why No 2nd Dose**

**Start of Block: 2nd Vaccine Questions**

Q30 Following **your second** COVID-19 vaccine, how would you rate the severity of these side effects on the following scale?

| None: No symptoms (1) | | Mild: Did not interfere with activities (2) | Moderate: Interfered with some activities (3) | Severe: Prevented regular daily activity (4) | Medical Care Required: ER, doctor, or hospital (5) |
| --- | --- | --- | --- | --- | --- |
| Arm soreness (1) | o | o | o | o | o |
| Muscle pain/ joint pain (25) | o | o | o | o | o |
| Headache (26) | o | o | o | o | o |
| Fever (99+ degrees ) (27) | o | o | o | o | o |
| Chills (28) | o | o | o | o | o |
| Nausea (29) | o | o | o | o | o |
| Vomiting (30) | o | o | o | o | o |
| Diarrhea (31) | o | o | o | o | o |
| Fatigue (32) | o | o | o | o | o |
| Redness around injection site (33) | o | o | o | o | o |
| Skin Rash around injection site (34) | o | o | o | o | o |
| Itching around injection site only (35) | o | o | o | o | o |

| Generalized itching (36) | o | o | o | o | o |
| --- | --- | --- | --- | --- | --- |
| Swollen lymph glands (37) | o | o | o | o | o |
| Allergic reaction (last in this set, proceed after answering) (38) | o | o | o | o | o |

**End of Block: 2nd Vaccine Questions**

**Start of Block: Other 2nd**

Q35 Did you experience any other symptoms not already listed with **your second** Covid-19 vaccine?

o Yes I did. (Please describe below.) (2)

o No (1)

**End of Block: Other 2nd**

**Start of Block: Second Vaccine Additional Qs**

Q23 Did you take any medications (either acetaminophen or a non-steroidal anti-inflammatory agent, such as ibuprofen) to treat the side effects of **your second** COVID-19 vaccine?

o Yes (2)

o No (1)

Q24 How many days of work or regular activities did you miss as a result of the side effects from your second COVID-19 vaccine?

o zero days (1)

o 1 day (2)

o 2 days (3)

o 3 days (4)

o 4 days or more (5)

**End of Block: Second Vaccine Additional Qs**

**Start of Block: Past Reactions**

Q21 Please rank the severity of any side effects you may have experienced with the below vaccines in the past.

| I've not had this vaccine. (6) | | None: No symptoms (1) | Mild: Did not interfere with activities (2) | Moderate: Interfered with some activities (3) | Severe: Prevented regular daily activity (4) | Medical Care Required: ER,  doctor, or hospital (5) | I don't know. (8) |
| --- | --- | --- | --- | --- | --- | --- | --- |
| Flu vaccine (1) | o | o | o | o | o | o | o |
| Pneumonia vaccine (2) | o | o | o | o | o | o | o |
| Tetanus (3) | o | o | o | o | o | o | o |
| Shingles (last in this set, proceed after answering) (4) | o | o | o | o | o | o | o |

**End of Block: Past Reactions**

**Start of Block: COVID Yes or No**

Q27 Had you ever tested positive for COVID-19 prior to receiving your first COVID-19 vaccine?

o Yes (1)

o No (2)

**End of Block: COVID Yes or No**

**Start of Block: COVID POSITIVE**

Q26 How long before your first vaccine did you test positive?

o I tested positive less than 30 days prior to my first COVID-19 vaccination (1)

o I tested positive 31-60 days prior to my first COVID-19 vaccination (2)

o I tested positive 61-90 days prior to my first COVID-19 vaccination (3)

o I tested positive more than 90 days prior to my first COVID-19 vaccination (4)

**End of Block: COVID POSITIVE**

**COVID Vaccine Side Effects: Survey Flow**

| **Standard: CONSENT (2 Questions)** | |
| --- | --- |
| **Branch: New Branch If**  **If After reading the consent form, do you agree to participate in this study? No. Is Selected** | |
|  | **Block: Enter to Win (1 Question)** |
|  | **EndSurvey:** |
| **Block: Age (1 Question) Standard: Gender (1 Question)**  **Standard: Race/Ethnicity (1 Question) Standard: Vaccinated? (1 Question)** | |
| **Branch: New Branch If**  **If Have you received a COVID-19 vaccine? No Is Selected** | |
|  | **Block: No Vaccine (1 Question) Block: Enter to Win (1 Question)** |
|  | **EndSurvey:** |
| **Standard: Vaccine WHY (1 Question) Standard: Vaccine Phase (1 Question) Standard: Brand (1 Question)** | |
| **Branch: New Branch If**  **If Which brand of vaccine did you receive? I'm not sure Is Selected** | |
|  | **Block: Enter to Win (1 Question)** |
|  | **EndSurvey:** |
| **Branch: New Branch If**  **If Which brand of vaccine did you receive? Johnson & Johnson (also referred to as Janssen) Is Selected** | |
|  | **Standard: J&J Vaccine Rxn (1 Question) Standard: Other JJ (1 Question)**  **Standard: J&J Additional Qs (2 Questions)** |
| **Branch: New Branch** | |

| **If**  **If Which brand of vaccine did you receive? Moderna Is Selected Or Which brand of vaccine did you receive? Pfizer Is Selected** | | |
| --- | --- | --- |
|  | **Standard: Both Doses? (Moderna/Pfizer) (1 Question) Standard: 1st Vaccine Rxn (1 Question)**  **Standard: Other 1st (1 Question)**  **Standard: 1st Vaccine Additional Qs (2 Questions)** | |
|  | **Branch: New Branch If**  **If Have you received BOTH doses of the vaccine? No Is Selected** | |
|  | | **Block: Why No 2nd Dose (1 Question)** |
|  | **Branch: New Branch If**  **If Have you received BOTH doses of the vaccine? Yes Is Selected** | |
|  | | **Block: 2nd Vaccine Questions (1 Question) Standard: Other 2nd (1 Question)**  **Standard: Second Vaccine Additional Qs (2 Questions)** |
| **Block: Past Reactions (1 Question) Block: COVID Yes or No (1 Question)** | | |
| **Branch: New Branch If**  **If Had you ever tested positive for COVID-19 prior to receiving your first COVID-19 vaccine? Yes Is Selected** | | |
|  | **Block: COVID POSITIVE (1 Question) Standard: Enter to Win (1 Question)** | |
|  | **EndSurvey:** | |
| **Branch: New Branch If**  **If Had you ever tested positive for COVID-19 prior to receiving your first COVID-19 vaccine? No Is Selected** | | |
|  | **Standard: Enter to Win (1 Question)** | |
|  | **EndSurvey:** | |

Page Break
